# Factors Associated with Insomnia and Aggression among Healthcare Workers during COVID-19 Pandemic

**DOI:** 10.3390/ijerph20021433

**Published:** 2023-01-12

**Authors:** Anna Maria Cybulska, Agnieszka Weymann, Kamila Rachubińska, Szymon Grochans, Grzegorz Wójcik, Elżbieta Grochans

**Affiliations:** 1 Department of Nursing, Faculty of Health Sciences, Pomeranian Medical University in Szczecin, 48 Żołnierska St., 71-210 Szczecin, Poland; 2 Independent Clinical Public Hospital No. 2 in Szczecin, Pomeranian Medical University in Szczecin, Powstancow Wielkopolskich 72, 72-111 Szczecin, Poland; 3 Department of Clinical Nursing, Faculty of Health Sciences, Pomeranian Medical University in Szczecin, 48 Żołnierska St., 71-210 Szczecin, Poland

**Keywords:** COVID-19, insomnia, aggression, healthcare workers

## Abstract

(1) Healthcare workers are exposed to increased risks of insomnia and aggression during the COVID-19 pandemic. The aim of the study was to assess insomnia, sleep disturbances, and aggression and identify the associated risk factors among healthcare workers during the COVID-19 pandemic. (2) A total of 264 healthcare workers participated in the study. The study was conducted with the diagnostic survey method, using the Buss–Perry Aggression Questionnaire, the Athens Insomnia Scale, the Pittsburgh Sleep Quality Index, and a self-administered questionnaire. (3) The vast majority of the respondents (81.06%) suffered from insomnia and had poor sleep quality (78.03%). Education (*p* = 0.038), marital status (*p* = 0.043), and working with patients suffering from COVID-19 (*p* = 0.024) were statistically significant contributors to insomnia. Age was found to significantly correlate with total aggression (r = −0.133 *p* = 0.031), verbal aggression (r = −0.138 *p* = 0.025), and anger (r = −0.151 *p* = 0.014). The analysis demonstrated statistically significant relationships between gender and physical aggression (*p* = 0.017), anger (*p* = 0.032), and hostility (*p* = 0.002). A statistically significant positive correlation between the quality of sleep as per the PSQI and all subscales of the BPAQ was found (*p* < 0.001). (4) A considerable proportion of HCWs experienced sleep disturbances during the outbreak, stressing the need to establish ways to reduce long-term adverse outcomes associated with chronic insomnia and mental health problems and adjust interventions under pandemic conditions.

## 1. Introduction

The Coronavirus-Disease-2019 (COVID-19) pandemic caused by severe acute respiratory syndrome coronavirus 2 (SARS-CoV-2) has been recognized as a public health emergency of international concern. The World Health Organization (WHO) expressed concern regarding the impact of COVID-19 on mental health along with psychosocial and socioeconomic considerations of the pandemic [1].

Healthcare workers are exposed to increased risks of insomnia and aggression during the COVID-19 pandemic. In a pandemic situation, with an increased number of employees under heavy workloads, the exposure to negative psychological effects is even greater, and that, in turn, can increase the level of anxiety, depression, or stress [2]. It is important to emphasize that good-quality sleep promotes better health and wellbeing, is conducive to quicker regeneration of the body, relieves work fatigue, and allows for maintaining physical strength [3,4]. Data from the literature indicate numerous factors contributing to insomnia and psychological problems. The most important are female gender, a younger age group (<40 years old), the presence of chronic/psychiatric illnesses, unemployment, healthcare profession, residence in rural areas, the risk of contact with patients infected with COVID-19, and isolation [5].

As a public health emergency of international concern, the COVID-19 pandemic is a traumatic event affecting both sleep and the mental health of the general population and healthcare providers [5,6,7]. Poor-quality sleep may impair personnel’s attentiveness and decision-making ability, consequently decreasing clinical performance, which, in turn, can negatively affect the quality of care [8]. Moreover, sleep disturbances can contribute to the development of various problems related to the psychological health of healthcare workers such as depression or anxiety and have a long-term effect on their health [9]. Owing to the significant negative effect of sleep disturbances on healthcare employees, it is vital to employ an early intervention with the purpose of minimizing the risk of mental illnesses.

Jahrami et al. [10] demonstrated that the global pooled prevalence rate of sleep problems during the COVID-19 pandemic amounted to 35.7%, with the highest percentage observed in COVID-19 patients (74.8%), followed by healthcare workers (36%). Alimoradi et al. [11] estimated a similar prevalence of sleep disturbances in the general population to be 31%, which is in line with the results of other studies [12]. Al Maqbali et al. [13] reported that a slightly higher percentage was observed among nurses (43%) in comparison with other healthcare employees.

Frontline healthcare workers (FHCW), understood as medical personnel (doctors, nurses, medical students, technicians, respiratory therapists, etc.) involved in the assessment, quarantine, isolation, and treatment of COVID-19 patients are particularly at risk of stressors [14].

Hiong et al. [15] demonstrated that relatively high levels of anxiety symptoms (from 6.33% to 50.9%), depression (14.6–48.3%), posttraumatic stress disorder (PTSD) (7–53.8%), and mental distress (34.43–38%) in the general global population during the COVID-19 pandemic had an effect on the risk of sleep disturbances.

Barua et al. [16] report that, among the studied doctors, 18.6% suffered from insomnia; additionally, 36.5% suffered from anxiety, 38.4% from depression, and 31.9% from fear of COVID-19; however, insufficient resources in the workplace was the most significant predictor across all psychological results. In turn, Qi et al. [6] demonstrated that frontline healthcare workers (FHCW) reported markedly lower sleep quality and more frequent occurrences of sleep disturbances, insomnia, and depression in comparison with personnel not involved in the frontline management of COVID-19. Wang et al. [17] also confirmed that the prevalence of sleep disturbances was higher among FHCW in comparison with non-FHCW employees and non-medical personnel, whereas anxiety and depression were found in all groups. These findings are in line with the results of other studies [18,19].

Workplace violence (WPV) against medical personnel is a complex issue., and rigorous research that would offer a solution is lacking [20].

According to reports from the World Health Organization (WHO), approximately 8–38% of medical personnel had experienced physical violence before the COVID-19 pandemic [21]. Additionally, in the wake of the COVID-19 pandemic, increasing distrust on the part of the general public towards specialists has been reported. Consequently, there has been increased reporting of acts of violence towards medical personnel (verbal or physical aggression) from patients, family, or co-workers [22].

Aggression in the workplace is a common phenomenon, but there are professional groups that are particularly exposed to it, for example, the medical profession, including the professional group of nurses. In these groups, the source of aggression can be patients as well as colleagues [23]. Aggression in the workplace can take different forms, therefore it is vital to differentiate between physical and verbal aggression. Physical aggression refers to the use of physical force against another person, which can result in physical and psychological harm (e.g., beating, kicking, slapping, pushing, biting, or pinching). Verbal aggression includes all forms of verbal violence such as verbal abuse, bullying, swearing, and derogatory language. Though the terms “aggression” and “violence” are often used interchangeably, workplace violence is a specific form of aggression, which takes place at the workplace and is intended to cause physical harm [24].

Physical and verbal aggression from patients, their next of kin, and visitors is a widespread problem in healthcare facilities and has become a major public health issue. Nursing is one of the professions most exposed to aggression and violence [25,26].

During the COVID-19 pandemic, an increased frequency of violence towards healthcare workers was reported [27]. This has been a problem for many years [19]; however, with the limited access to healthcare services during the pandemic, there has been a significant increase in aggressive behavior towards FHCW [19], such as physical and psychological violence and vicarious violence [28]. The meta-analysis conducted by Li et al. in 2020 shows the prevalence of violence in the workplace at 19.3% [29] as well as a higher prevalence of acts of violence towards nursing personnel than towards doctors. Experiencing violence at work is associated with numerous negative health effects such as loss of self-esteem, sleep disturbances, anxiety, depression, irritability, or difficulty concentrating; it can also result in decreased motivation and commitment of employees or lead to professional burnout syndrome [30]. It is worth noting that the pace and intensity of work and interpersonal conflicts are connected to an increase in aggressive behavior at work [31]. Therefore, it is crucial to prevent violence at work by implementing increased preventive measures aimed at ensuring the wellbeing of medical personnel and care of their physical and mental health [32].

The pandemic is evolving rapidly, hence there is a need to gather data allowing a global review of the mental health of healthcare workers during the COVID-19 pandemic [33,34,35,36]. The immediate priority is to monitor rates of mood, sleep, and other mental health issues in order to understand the mediating factors and form tailored interventions [37]. The aim of the study was to assess insomnia, sleep disturbances, and aggression and identify the associated risk factors among healthcare workers during the COVID-19 pandemic.

We hypothesized that:

- There is a relationship between the occurrence of insomnia, sleep disturbance, and increased aggression among medical personnel during the COVID-19 pandemic.

## 2. Materials and Methods

### 2.1. Settings and Design

The research was conducted in 2021 on healthcare workers working directly with patients diagnosed with SARS-CoV-2 infection in the Independent Public Clinical Hospital No. 1 of the Pomeranian Medical University named after Prof. Tadeusz Sokołowski in Szczecin.

The inclusion criteria were age >18 years, a current license to practice as a nurse, having worked a minimum of 3 months in wards admitting patients diagnosed with SARS-CoV-2, and informed consent to participate in the study.

The size of the study sample was established by a statistician on the basis of statistical data concerning the number of healthcare workers working in the Independent Public Clinical Hospital No. 1 of the Pomeranian Medical University named after Prof. Tadeusz Sokołowski in Szczecin. The confidence level was set at 95%, the maximum error at 7%, and the estimated fraction size at 0.5. A total of 300 individuals were invited to participate in the study; however, due to staff shortages and workload, 264 individuals were enrolled in the study.

The study was carried out using a traditional method of distributing paper questionnaires by an interviewer among healthcare workers employed in the hospital. At every stage of the research, the respondents had the opportunity to obtain assistance if needed, as well as withdraw from the study.

The study was conducted according to the guidelines of the Declaration of Helsinki and approved by the Ethical Committee of the Pomeranian Medical University in Szczecin by Resolution No. KB-0012/25/04/2020/Z. The study was conducted taking into account ethical considerations. Informed consent was required, and participation in the study was voluntary. Moreover, the participants were assured of anonymity and confidentiality and were free to withdraw from the study at any stage.

### 2.2. Research Instruments

The following standardized survey instruments were used:

- The Buss–Perry Aggression Questionnaire (BPAQ) is a measure of aggression in adults. It consists of 29 items, subdivided into four factors: Physical aggression (9 items), verbal aggression (5 items), anger (8 items), and hostility (8 items) [27]. The BPAQ sub-scales include different numbers of questions, therefore each scale shows a different value range. However, for each range, a higher score means a greater intensity of a given type of aggressive tendency. There are no norms that allow one to determine the exact score that would mark “great intensity”. Nevertheless, for each subscale, it is possible to calculate the mean score for a question and interpret it according to the Key as per individual question—where (having reversed the score in respective questions) 1 would indicate “definitely not”, 2 “rather not”, 3 “difficult to say”, 4 “rather yes”, and 5 “definitely yes”. Cronbach’s alpha for the BPAQ is 0.89, for physical aggression is 0.85, for verbal aggression is 0.72, for anger is −0.83, and for hostility is −0.77.
- Athens Insomnia Scale (AIS) includes 8 questions and is a common and easily interpretable screening tool used to measure insomnia, with a total score ranging from 0 to 24 points. The first five items relate to sleep-related symptoms and correspond to criterion A of the ICD-10 diagnosis of inorganic insomnia. If a given symptom occurred at least three times a week during a one-month period, it is to be marked –, which is consistent with the duration and frequency of symptoms required for the ICD-10 diagnosis of insomnia (criterion B). The remaining three items refer to daytime functioning (mood, physical and mental performance, sleepiness) and correspond to criterion C of the ICD-10 diagnosis of insomnia, which includes complaints about insomnia consequences experienced during the day. Cronbach’s alpha for the AIS is 0.90 [38].
- The Pittsburgh Sleep Quality Index (PSQI) is a self-rated questionnaire used for the assessment of the quality of sleep and sleep disturbances over a 1-month time interval. Nineteen individual items generate seven “component” scores, namely, subjective sleep quality, sleep latency, sleep duration, habitual sleep efficiency, sleep disturbances, use of sleeping medication, and daytime dysfunction. The sum of scores for the seven components yields one global score. The PSQI has a sensitivity of 89.6% and specificity of 86.5% for identifying cases with a sleep disorder, using a cut-off score of 5. Cronbach’s alpha for the PSQI is 0.83 [39].
- Self-administered questionnaire—includes questions about sociodemographic data (age, marital status, parental status, place of residence, and education), employment (ward, employment), history of exposure to COVID-19, and additional information required in relation to COVID-19 collected using the authors’ questionnaire.

### 2.3. Statistical Analysis

The analysis of quantitative variables was performed by calculating the mean, standard deviation, median, quartiles, minimum, and maximum values. The comparison of quantitative variables in two groups was performed with the use of the Mann–Whitney U test. The correlations between quantitative variables were analyzed with the Spearman correlation coefficient. The Kruskal–Wallis test and Dunn test were used in this study.

All calculations were performed using R version 4.1.2. (RStudio, Boston, MA, USA). The level of statistical significance was set at *p* < 0.05 [40].

## 3. Results

### 3.1. Characteristics of the Respondents

The study sample consisted of 264 healthcare workers. The mean age of the respondents was 43.42 years (SD = 11.05). The vast majority of the respondents were female (82.58%), in a formal relationship (54.92%), with higher education attainment (68.56%), and were living in a city of more than 100,000 residents (43.56%) and working at a hospital (46.97). More than half of the respondents had children (72.35%), and 84.85% of healthcare workers were in close contact with a person with a confirmed COVID-19 infection.

### 3.2. Analysis of the Severity of Insomnia and Aggression among Healthcare Workers during the SARS-CoV-2 Pandemic

The analysis was performed regarding sleep disorders (according to the AIS and PSQI) and aggression (according to the BPAQ) among healthcare workers during the SARS-CoV-2 Pandemic.

The vast majority of the respondents (81.06%) suffered from sleep disorders and had poor sleep quality (78.03%) (Table 1).

The analysis of the results obtained with the BPAQ showed that the mean score for total aggression amounted to 70.18 points, while physical aggression had 18.96 points, verbal aggression had 13.77 points, anger had 18.78 points, and hostility had 18.67 points (Table 2).

### 3.3. Analysis of the Relationship between Sociodemographic Variables (Age, Education, Place of Residence, Marital Status) and Sleep Disorder and Aggression among Healthcare Workers during the SARS-CoV-2 Pandemic

This study analyzed the influence of selected sociodemographic variables (age, education, marital status, and parental status) on sleep disorder and aggression among healthcare workers during the SARS-CoV-2 Pandemic.

It was found that insomnia was significantly more pronounced in respondents who were in a formal relationship rather than those that were single (*p* = 0.043). Moreover, the study determined that insomnia was significantly less pronounced in childless respondents in comparison with other respondents (*p* = 0.004). Insomnia and trouble sleeping were found to be significantly more common in respondents who had completed Higher education (Bachelor) as compared with other respondents (*p* = 0.038 and *p* = 0.002, respectively).

There were no statistically significant correlations between age and insomnia or sleep quality (Table 3). The analysis of the influence of sociodemographic variables (gender, marital status) on insomnia or sleep quality among healthcare workers during the SARS-CoV-2 pandemic did not reveal any statistically significant differences (Table 4).

Age was found to significantly correlate with total aggression (r = −0.133 *p* = 0.031), verbal aggression (r = −0.138 *p* = 0.025), and anger (r = −0.151 *p* = 0.014). There were no statistically significant correlations between age and physical aggression or hostility (Table 4). The analysis demonstrated statistically significant relationships between gender and physical aggression (*p* = 0.017), anger (*p* = 0.032), and hostility (*p* = 0.002). It was found that in women, anger and hostility were more pronounced, and in men, we observed physical aggression. The analysis of the data did not demonstrate the influence of gender on total aggression and verbal aggression (Table 5). Marital status, parental status, and education were found to show significant differences regarding physical aggression (*p* = 0.032). Physical aggression was significantly more pronounced in respondents in a formal relationship rather than single participants and in respondents with underage children rather than childless respondents. In turn, all types of aggressive tendencies were found to be significantly more pronounced in employees who completed Higher education (Bachelor) in comparison with other groups (Table 5).

### 3.4. Analysis of the Relationship between Work-Related Variables (Work Experience, Contact with a Patient Diagnosed with COVID-19) on Sleep Disorder and Aggression among Healthcare Workers during the SARS-CoV-2 Pandemic

This study analyzed the influence of selected work-related factors (work experience, place of work, and contact with COVID-19 patients) on sleep disorder and aggression among healthcare workers during the SARS-CoV-2 Pandemic.

On the basis of the data collected, it was possible to demonstrate a statistically significant negative correlation between work experience and Total aggression (r = −0.138 *p* = 0.025), verbal aggression (r = −0.139 *p* = 0.023), and anger (r = −0.165 *p* = 0.007). Moreover, the analysis showed a statistically significant positive correlation between work time and physical aggression (r = 0.168 *p* = 0.006) and anger (r = 0.121 *p* = 0.05). Additionally, there was a statistically significant negative correlation between work time and verbal aggression (r = −0.132 *p* = 0.032) (Table 6).

Work time was found to significantly correlate with insomnia (r = 0.124 *p* = 0.044). The analysis showed a statistically significant relationship between the place of work (*p* = 0.002) and insomnia, according to AIS. Insomnia was markedly more pronounced in healthcare workers in non-invasive treatment wards, surgical wards, or highly specialized hospital wards in comparison with those working in other wards. Work experience and employment types were not statistically significant contributors to insomnia according to the AIS (Table 7).

The study shows a statistically significant relationship between total aggression, verbal aggression, anger, hostility, and place of work. Total aggression was markedly more pronounced in healthcare workers of surgical wards in comparison with those employed in other wards and, additionally, among respondents working in highly specialized wards, it was found to be significantly more pronounced than in respondents employed in non-invasive treatment wards (*p* <0.001). Verbal aggression was significantly more intensified in healthcare workers of surgical and highly specialized wards rather than in those working in non-invasive treatment wards (*p* = 0.037). Anger was found to be significantly more pronounced in respondents working in surgical wards than in those working in highly specialized wards who, in turn, showed a markedly higher level of anger in comparison with the employees of the remaining types of wards under study (*p* < 0.001). Hostility was significantly more intensified among surgical-ward employees compared to the remaining groups (*p* < 0.001) (Table 8).

### 3.5. Analysis of the Correlation between Aggression and Sleep Disorder among Healthcare Workers during the SARS-CoV-2 Pandemic

On the basis of the data obtained, a statistically significant positive correlation between insomnia as per AIS with all aspects of aggression according to BPAQ was found. This implies that the greater intensity of insomnia, the greater the intensity of all types of aggressive tendencies (*p* < 0.001) (Table 9).

Moreover, a statistically significant positive correlation between the quality of sleep as per PSQI and all subscales of BPAQ was found. This can be interpreted as the greater the intensity of sleep problems, the greater the intensity of all types of aggressive tendencies (*p* < 0.001).

## 4. Discussion

### 4.1. Analysis of the Severity of Insomnia and Aggression among Healthcare Workers during the SARS-CoV-2 Pandemic

The review of the literature indicates a high incidence of aggression or sleep disorders among healthcare workers. Therefore, during the COVID-19 pandemic, serious mental health problems among healthcare workers and potential health crises among this group have become even more apparent [41].

The prevalence of clinical insomnia (13–15%) has increased since the start of the pandemic (compared to 6–10% for pre-pandemic chronic insomnia). These data are consistent with other research that has also shown greater insomnia prevalence rates during the post-pandemic period, though some studies have shown even larger increases in moderate-to-severe insomnia symptoms [42,43,44,45]. On the grounds of our own studies, it was found that the vast majority of the respondents suffered from sleep disorders and had poor sleep quality.

The meta-analysis by Pappa et al. [35] demonstrated that the prevalence of insomnia was 34.32%; in turn, the meta-analysis by Mervaldi et al. [41] estimated the prevalence of sleep disorders at 44% and was found to be predominant in women.

Fu et al. [46] report that the global prevalence of insomnia assessed with the Athens Insomnia Scale (AIS) was 30%. In turn, according to Jahrami et al. [10], the global pooled prevalence rate of sleep disorders in the general population was 32.3%, whereas the total prevalence of sleep disorders among healthcare workers was found to be slightly higher, though comparable (36.0%). According to Bozan et al. [47], sleep quality, assessed with PSQI, deteriorated after having been infected with COVID-19 as compared with the pre-infection period (*p* < 0.001), thus suggesting that the decrease in quality may be a psychosomatic consequence of the COVID-19 infection.

Our own studies have demonstrated that it is important to conduct further research assessing the prevalence of aggression among medical personnel. The review of the literature on the subject shows that during the COVID-19 pandemic, there were reports of violence, bullying, or stigmatization against healthcare workers and patients [48]. There is evidence that the level of aggression during the COVID-19 pandemic has increased. Approximately 65.5% of healthcare workers reported exposure to violence at the workplace, predominantly verbal violence [49]. Another study indicates that caregivers and nursing assistants were more exposed to violence during the pandemic [50]. During the COVID-19 pandemic, approximately 44.4% of nurses experienced physical violence and 67.8% experienced verbal violence. It was found that nurses providing care to a patient diagnosed with COVID-19 had 2.18- and 2.10-times higher risk of physical and verbal violence, respectively [51]. All the above-mentioned evidence is based on type II aggression, i.e., aggression towards healthcare workers. However, despite the evidence, the information on aggression from nurses directed towards others is lacking, particularly with reference to the COVID-19 pandemic [52]. In turn, the studies by Liu et al. [53] conducted in 2019 demonstrated that 62% of medical personnel reported any form of violence experienced in professional work. The studies by Hamzaoglu [54] demonstrated that healthcare workers experience verbal violence much more frequently than physical violence. It is difficult to determine the exact epidemiology of episodes of violence against frontline healthcare workers during the COVID-19 pandemic, yet numerous reports show different forms of violence [55] and discrimination against certain populations during that period [56,57].

### 4.2. Analysis of the Relationship between Sociodemographic Variables (Age, Education, Place of Residence, Marital Status) on Sleep Disorder and Aggression among Healthcare Workers during the SARS-CoV-2 Pandemic

According to our own research, education, marital, and parental status were statistically significant contributors to insomnia according to the AIS. The studies demonstrated that insomnia was markedly more pronounced in respondents in a formal relationship as compared with single respondents, and it was also found to be higher in respondents who had completed Higher education (Bachelor) in comparison with other respondents. Furthermore, it was determined that insomnia was significantly less pronounced in childless respondents as compared with the rest of the group. Education was a statistically significant contributor to sleep quality according to PSQI. Sleep disturbances were markedly more pronounced among respondents who completed Higher education (Bachelor) as compared with other respondents.

The review of the literature shows that there are numerous factors affecting the occurrence of sleep disturbances among healthcare workers, for example, providing care to patients diagnosed with COVID-19 [58], being a frontline healthcare worker [59], work schedule [60], shift work [61], older age of the respondents, and female gender [62].

Age was found to significantly correlate with total aggression, verbal aggression, and anger. There were no statistically significant correlations between age and physical aggression or hostility. The analysis demonstrated statistically significant relationships between gender and physical aggression, anger, and hostility. It was found that in women, anger and hostility were more pronounced, whereas, in men, we observed physical aggression. Marital status, parental status, and education were found to significantly correlate with physical aggression. Physical aggression was markedly more pronounced in respondents in a formal relationship, as compared with single respondents, and in those who had underage children in comparison with childless respondents. In turn, all types of aggressive tendencies were significantly greater among respondents who completed Higher education (Bachelor) in comparison with the other groups. The literature review demonstrates that older age [63], female gender [64,65], nursing profession [66], and working in a ward taking in COVID-19 patients [67,68] can constitute risk factors for poor sleep quality and insomnia among healthcare workers during the COVID-19 pandemic. The study by Molero et al. [27] observed that nurses employed in the healthcare system were predominantly exposed to violence. Moreover, nurses who had been attacked scored higher in terms of emotional exhaustion and depersonalization, and most of the respondents considered COVID-19 to be an important factor in the increase in violence towards healthcare workers. In their study, Gacki-Smith et al. [69] demonstrated that violence was significantly associated with having less experience and younger age, which most likely stems from respect towards older members of the healthcare system on the part of the general public. Moreover, the vast majority of the respondents did not report incidents of violence to their superiors. Lafta et al. [70] show that violence primarily affects younger age groups (<40 years of age) and women. This most likely results from the perception of female healthcare workers as weaker and more incapable of defending themselves. The review of the literature indicates numerous factors contributing to violence in the workplace, such as heavy workload, high expectations of patients, substance abuse by patients, long waiting period, refusing unacceptable demands, sensational media reports, and inadequate protective measures [71]. Most studies concern aggression against healthcare workers. Despite the aforementioned evidence, information concerning aggression expressed by nurses towards others is lacking.

### 4.3. Analysis of the Relationship between Medical Variables on Sleep Disorder and Aggression among Healthcare Workers during the SARS-CoV-2 Pandemic

Our own studies demonstrated that work time significantly correlated with insomnia. Moreover, there was a statistically significant relationship between insomnia and working with COVID-19 patients and between sleep quality measured with the PSQI and working with patients diagnosed with COVID-19. The studies did not reveal any statistically significant relationships between sleep quality and work-related variables among healthcare workers during the SARS-CoV-2 pandemic. On the basis of the collected data, a statistically significant relationship between work experience, total aggression, verbal aggression, and anger was shown. Additionally, a statistically significant correlation between work time, physical aggression, and anger was found, as well as a statistically significant negative correlation between work time and verbal aggression. The studies show a statistically significant relationship between total aggression, verbal aggression, anger, hostility, and the place of work. Moreover, a statistically significant relationship between verbal aggression and working with COVID-19 patients was identified. On the basis of the obtained results, a statistically significant positive correlation between insomnia according to AIS with all aspects of aggression according to BPAQ was found. Moreover, a statistically significant positive correlation between the quality of sleep according to PSQI and all subscales of aggression according to BPAQ was demonstrated. This means that more pronounced sleep disorders translate into a higher intensity of all types of aggressive tendencies.

The review of the literature on the subject shows that during the COVID-19 pandemic, violence in the workplace was found to increase, which presumably contributes to the increased prevalence rate of sleep disorders among healthcare workers [72,73,74]. Nevertheless, it is not entirely clear whether improved sleep quality would directly translate into decreased mental stress among healthcare workers.

Meaklim et al. [75] demonstrated that people experiencing the symptoms of insomnia during the pandemic reported more severe symptoms of depression, anxiety, and stress as compared with the people who experienced symptoms of insomnia before the outbreak. The treatment of insomnia is of particular importance since sleep disorders occurring during the COVID-19 pandemic increase the risk of long-term negative psychological effects [76].

The study by Demichelis et al. [77] points to a significant relationship between sleep and aggression in the general population. It was found that the experimental reduction of sleep leads to increased aggression. This means that worse sleep quality is associated with and results in aggression. Moreover, studies by Van Veen et al. [78] confirm that poor sleep quality is consistently associated with increased levels of aggression. There are studies indicating the relationship between worse sleep quality and increased aggression; however, there are also studies showing some discrepancies and others reporting no significant relationship between the said factors [79,80].

Killgore et al. [49] found that an elevated level of aggression was predominantly determined in people in isolation as compared with those who reported not being under such limitations. Moreover, this pattern was manifested in all subscales of the BPAQ. This means that during the COVID-19 pandemic, people in isolation were more likely to lose their temper and attack others physically as well as verbally. The findings are in line with the frustration–aggression hypothesis, which stipulates that being prevented from achieving a desired goal results in a negative affective state, which, in turn, translates into a tendency towards aggression [49]. Numerous studies suggest that the COVID-19 pandemic had a significant effect on the accumulation of various factors aggravating aggression and was manifested as violence in both the general population and in healthcare works [81,82,83,84].

The meta-analysis by Yosep et al. [85] determined that nursing staff are bullied in the workplace through physical and verbal aggression. The resulting physical effects of the abuse include problems falling asleep, vertigo, and palpitations [86]. The social effects include a lack of self-confidence, low self-esteem, anger, a feeling of helplessness, and sadness [87]. Additionally, Chakraborty et al. [88] observed that violence towards healthcare workers is a global public health issue.

Pagnucci et al. [89] demonstrated numerous predictors of violence, such as the characteristics of both patients and nurses (e.g., gender, age, education [90], poor training of medical personnel [91], and lack of communication between the personnel and patients [92]. All the aforementioned variables have a significant effect on the increase in aggression and violence [93,94].

In conclusion, problems with sleep or aggression seem to be widespread during the COVID-19 pandemic. The vast majority of respondents experienced sleep disturbances during the pandemic. Therefore, more longitudinal and randomized controlled studies are needed to examine how insomnia and the expression of aggression change over time and determine the causal link between problems sleeping and mental distress.

## 5. Implications

- It is necessary to establish and implement screening programs and preventive measures concerning sleep disorders to help healthcare workers identify and overcome sleep disturbances. They must be encouraged to apply evidence-based strategies, e.g., cognitive-behavioral therapy, meditation, sports, and healthcare interventions. Moreover, medical personnel should be trained in identifying and treating sleep problems in various populations.
- Studies should be conducted with the aim of determining the extent of insomnia by its severity; it is necessary to conduct longitudinal studies to determine whether insomnia is short-term or long-term.
- To ensure the wellbeing of healthcare workers and quality of care during a pandemic, it is vital to provide targeted preventive measures and psychological support to the group. Additionally, effective programs for fighting aggression may have a positive effect on the sleep of healthcare workers.
- Good-quality sleep is of fundamental importance to safety in the workplace and the health of healthcare workers. By means of assessing sleep quality during times of crisis, medical personnel can identify possible means of promoting health and safety through education on sleep hygiene, monitoring sleepiness or fatigue, and the assessment of the possibility of changing organizational policy.
- The COVID-19 pandemic has had a drastic effect on the functioning of healthcare in Poland. The physical and emotional load on healthcare workers is substantial and constitutes an additional risk to providing patient care and the productivity of a hospital. It is necessary to conduct further research on the relationship between workplace violence in medical facilities and pandemic-related factors. Aggression from patients is indeed an obstacle to ensuring the best practices and providing efficient care. It is a challenge that may delay treatment and allocation and inhibit the best possible result of hospital treatment. Therefore, it is vital to implement more safety precautions, reduce workplace violence, improve communication and problem-solving methods with respect to patient care, and introduce training courses with respect to the means of coping with aggression.

## 6. Limitation

The present study has some limitations.

- The study was conducted in one medical facility in Szczecin during the second wave of the pandemic, therefore the possibility of generalization of the obtained results may be limited.
- The data were collected with self-report questionnaires and not with clinical interviews.
- Objective sleep measurement data were not collected to confirm the subjective reports of sleep.
- One of the disadvantages of adopting a convenient sample was the small sample size. A larger study group would provide greater statistical power.

## 7. Conclusions

- A considerable proportion of HCWs have experienced sleep disturbances during the outbreak, stressing the need to establish ways to reduce long-term adverse outcomes associated with chronic insomnia and mental health problems and adjust interventions under pandemic conditions.
- Our study demonstrates a significant association between sociodemographic variables (age, gender, and marital and parental status) and work-related variables (work time and work experience, working with COVID-19 patients) and the prevalence of insomnia or aggression among healthcare workers during the COVID-19 outbreak.
- Insomnia and sleep disturbance were found to be connected to aggression. It transpires that sleep plays a significant role in aggressive behavior. Further studies are necessary to demonstrate the relationship between sleep disturbance and aggression, and to investigate the moderating and intervening variables that would explain when and in what way aggression affects sleep. Since nurses are subject to different types of aggression and experience sleep disturbance, it is vital to implement appropriate interventions to protect the mental health of nurses, not only during the pandemic, characterized by an increase in aggressive behavior and sleep disturbance but also on a regular basis. Positive attitudes towards work and well-rested personnel may provide better patient care and superior quality of service.

## Figures and Tables

**Table 1:** Sleep disorder of the respondents.

Severity Category			
Variables (Points)		*n*	%
AIS	No insomnia disorder	50	18.94
	Yes > 8 points	214	81.06
PSQI	Good sleep quality	58	21.97
	Poor sleep quality	206	78.03

AIS—Athens Insomnia Scale; PSQI—The Pittsburgh Sleep Quality Index.

**Table 2:** Aggression according to BPAQ.

BPAQ	M	SD	Average per Question	Me	Min–Max	Q1–Q3
Total aggression	70.18	17.14	2.42	71	38–117	57–84
physical aggression	18.96	6.13	2.11	18	9–37	13–25
verbal aggression	13.77	3.62	2.75	14	5–24	11–16
Anger	18.78	5.87	2.68	20	7–32	14–24
Hostility	18.67	6.11	2.33	19	8–39	13–24

M—mean; SD—standard deviation, Me—median, Min—minimum Max—maximum Q—quartile, BPAQ, The Buss–Perry Aggression Questionnaire.

**Table 3:** The influence of age on sleep disorder and aggression.

Variables (Points)		Age	
		r	*p*
AIS		0.022	0.725
PSQI		0.01	0.873
BPAQ	Total aggression	−0.133	0.031
	physical aggression	−0.085	0.169
	verbal aggression	−0.138	0.025
	Anger	−0.151	0.014
	Hostility	−0.089	0.149

AIS—Athens Insomnia Scale; PSQI—The Pittsburgh Sleep Quality Index; BPAQ—The Buss–Perry Aggression Questionnaire, *p*—significance level.

**Table 4:** The influence of selected sociodemographic variables on sleep disorder.

Variables		AIS		PSQI	
		M	SD	M	SD
Gender ^	Women (*n* = 218)	9.67	4.28	8.42	3.2
	Men (*n* = 46)	9.15	3.43	7.89	3.17
	*p*	0.366		0.468	
Marital status	Single—A (*n* = 74)	8.62	3.76	7.84	2.99
	Formal relationship—B (*n* = 145)	10.1	4.13	8.62	3.24
	Informal relationship—C (*n* = 45)	9.49	4.54	8.29	3.3
	*p* *	0.043 B > A		0.133	
Parental status	Childless—A (*n* = 73)	8.27	3.65	7.69	2.78
	with underage children—B (*n* = 82)	9.89	3.4	8.16	2.83
	with adult children—C (*n* = 109)	10.23	4.75	8.74	3.65
	*p*	0.004 C,B > A		0.35	
Education	secondary medical—A (*n* = 83)	9.19	4.69	8.29	3.53
	Higher education (Bachelor)—B (*n* = 116)	10.28	3.62	9.04	2.8
	Higher education (Master)—C (*n* = 65)	8.83	4.12	7.17	3.1
	*p* *	0.038 B > A,C		0.002 B > A,C	

M—mean; SD—standard deviation, *p*—significance level, ^ Mann–Whitney test, BPAQ, The Buss–Perry Aggression Questionnaire, * Kruskal–Wallis test + post-hoc analysis (Dunn’s test).

**Table 5:** The influence of selected sociodemographic variables on aggression.

Variables		Total Aggression		Physical Aggression		Verbal Aggression		Anger		Hostility	
		M	SD	M	SD	M	SD	M	SD	M	SD
Gender ^	Women (*n* = 218)	70.65	17.55	18.54	6.23	13.73	3.69	19.2	5.59	19.18	6.13
	Men (*n* = 46)	67.93	15.02	20.98	5.28	13.96	3.31	16.8	6.81	16.24	5.47
	*p*	0.351		0.017		0.431		0.032		0.002	
Marital status *	Single—A (*n* = 74)	67.08	18.52	17.66	6.38	13.58	3.9	17.81	6.02	18.03	6.24
	Formal relationship—B (*n* = 145)	72.19	16.34	19.71	6.08	13.73	3.45	19.63	5.58	19.12	5.95
	Informal relationship—C (*n* = 45)	68.8	16.78	18.67	5.6	14.22	3.72	17.64	6.25	18.27	6.42
	*p* *	0.059		0.032 B > A		0.433		0.063		0.559	
Parental status	Childless—A (*n* = 73)	69.85	19.07	17.51	6.44	14.03	4.14	19.16	6.13	19.15	7.2
	with underage children—B (*n* = 82)	69.94	16.15	20.34	5.63	13.52	3.14	18.61	5.88	17.46	5.43
	with adult children—C (*n* = 109)	70.58	16.63	18.89	6.1	13.79	3.61	18.65	5.73	19.25	5.71
	*p*	0.77		0.007 B > A		0.802		0.899		0.08	
Education *	secondary medical—A (*n* = 83)	63.4	18.22	17.55	6.48	12.47	3.56	16.67	5.73	16.43	6.46
	Higher education (Bachelor)—B (*n* = 116)	78.13	14.03	21.12	5.97	14.66	3.32	21.7	4.71	20.65	5.25
	Higher education (Master)—C (*n* = 65)	64.65	14.93	16.89	4.64	13.51	3.86	16.26	5.67	17.98	6.03
	*p* *	<0.001 B > C,A		<0.001 B > A,C		<0.001 B > C,A		<0.001 B > A,C		<0.001 B > C,A	

M—mean; SD—standard deviation, *p*—significance level, ^ Mann–Whitney test, *p*—significance level, BPAQ, The Buss–Perry Aggression Questionnaire, * Kruskal–Wallis test + post-hoc analysis (Dunn’s test).

**Table 6:** The effect of work experience and work time on sleep disorder and aggression.

Variables (Points)		Work Experience		Work Time	
		r	*p*	r	*p*
AIS		0.045	0.469	0.124	0.044
PSQI		0.011	0.856	−0.027	0.667
BPAQ	Total aggression	−0.138	0.025	0.068	0.272
	physical aggression	0.08	0.195	0.168	0.006
	verbal aggression	0.139	0.023	−0.132	0.032
	Anger	−0.165	0.007	0.121	0.05
	Hostility	−0.098	0.112	−0.057	0.354

AIS—Athens Insomnia Scale; PSQI—The Pittsburgh Sleep Quality Index, BPAQ—The Buss–Perry Aggression Questionnaire, *p*—significance level.

**Table 7:** The effect of the work-related variables on the occurrence of sleep disorders.

Variables		AIS		PSQI	
		M	SD	M	SD
Place of work	Non-invasive treatment ward—A (*n* = 33)	10.7	4.45	7.79	2.39
	Surgical ward—B (*n* = 71)	10.13	3.77	8.44	3.42
	Highly specialised ward—C (*n* = 124)	9.73	3.88	8.74	3.05
	Other—D (*n* = 36)	6.97	4.52	7.31	3.65
	*p*	0.002 A,B,C > D		0.056	
Type of employment	Employment contract (*n* = 192)	9.45	4.17	8.3	3.37
	No employment contract (*n* = 71)	9.93	4.11	8.49	2.7
	*p*	0.23		0.391	

AIS—Athens Insomnia Scale; PSQI—The Pittsburgh Sleep Quality Index, M—mean; SD—standard deviation, *p*—significance level.

**Table 8:** The effect of work-related variables on aggression.

Variables		Total Aggression		Physical Aggression		Verbal Aggression		Anger		Hostility	
		M	SD	M	SD	M	SD	M	SD	M	SD
Place of work	Non-invasive treatment ward—A (*n* = 33)	61.06	21.7	17.15	6.08	12.33	5	15.42	5.96	16.15	7.01
	Surgical ward—B (*n* = 71)	76.9	17.17	19.83	6.6	14.32	3.64	21.18	5.21	21.56	5.91
	Highly specialised ward—C (*n* = 124)	70.12	15.17	19.07	5.84	13.93	3.11	18.95	5.72	18.17	5.47
	Other—D (*n* = 36)	65.47	13.6	18.5	6	13.47	3.52	16.53	5.48	16.97	5.84
	*p*	<0.001 * B > C,D,A C > A		0.16		0.037 B,C > A		<0.001 B > C > D,A		<0.001 B > C,D,A	
Type of employment^	Employment contract (*n* = 192)	69.4	17.23	18.75	6.22	13.81	3.68	18.35	5.71	18.48	6.17
	No employment contract (*n* = 71)	72.11	16.9	19.41	5.86	13.63	3.49	19.87	6.23	19.2	5.99
	*p*	0.309		0.255		0.783		0.043		0.758	

M—mean; SD—standard deviation, *p*—significance level, ^ Mann–Whitney test *p*—significance level, BPAQ—The Buss–Perry Aggression Questionnaire, * Kruskal–Wallis test + post-hoc analysis (test Dunn’s test).

**Table 9:** The correlation of sleep disorder on intensity of aggression.

Variables (Points)		AIS		PSQI	
		r	*p*	r	*p*
BPAQ	Total aggression	0.329	<0.001	0.306	<0.001
	physical aggression	0.272	<0.001	0.214	<0.001
	verbal aggression	0.176	0.004	0.265	<0.001
	Anger	0.308	<0.001	0.295	<0.001
	Hostility	0.239	<0.001	0.219	<0.001

AIS—Athens Insomnia Scale; PSQI—The Pittsburgh Sleep Quality Index, BPAQ—The Buss–Perry Aggression Questionnaire, *p*—significance level.

## Data Availability

Not applicable.
